# Altered Cell Adhesion and Glycosylation Promote Cancer Immune Suppression and Metastasis

**DOI:** 10.3389/fimmu.2019.02120

**Published:** 2019-09-06

**Authors:** Heinz Läubli, Lubor Borsig

**Affiliations:** ^1^Laboratory for Cancer Immunotherapy, Department of Biomedicine and Medical Oncology, Department of Internal Medicine, University Hospital, Basel, Switzerland; ^2^Department of Physiology, University of Zurich, Zurich, Switzerland; ^3^Comprehensive Cancer Center, Zurich, Switzerland

**Keywords:** selectin, Siglec, integrin, immunity, sialic acid, tumor microenvironment

## Abstract

Cell-cell interactions and cell adhesion are key mediators of cancer progression and facilitate hallmarks of cancer including immune evasion and metastatic dissemination. Many cell adhesion molecules within the tumor microenvironment are changed and significant alterations of glycosylation are observed. These changes in cell adhesion molecules alter the ability of tumor cells to interact with other cells and extracellular matrix proteins. Three families of cell-cell interaction molecules selectins, Siglecs, and integrins have been associated with cancer progression in many pre-clinical studies, yet inhibition of cell adhesion as a therapeutic target is just beginning to be explored. We review how cell-cell interactions mediated by integrins and the glycan-binding receptors selectins and Siglec receptors support cancer progression. The discussion focuses on mechanisms during immune evasion and metastasis that can be therapeutically targeted by blocking these cell-cell interactions.

## Introduction

Cancer progression induces immune evasion and eventually metastasis, a process consisting of several steps enabling tumor cells to leave the primary tumor, to intravasate and survive in the circulation, to extravasate and seed in distant organs and to initiate growth of metastatic lesions. Tumor cell interactions with other cells in the environment contribute to immune evasion and metastasis at every step of this process. Adhesion molecules, on any cell, mediate interactions with other cells and the extracellular matrix in the microenvironment (1, 2). Since cell adhesion receptors are connected to signal-transduction pathways, these cell-cell and cell-matrix interactions modulate cell phenotype, proliferation, differentiation, survival, and migration. Consequently, changes in expression of cell adhesion molecules and their ligands directly affect immune evasion and metastasis.

Malignant transformation changes not only the expression of cell adhesion molecules but also causes profound changes in cell surface glycosylation (3, 4). Cancer-associated glycosylation promotes the interaction of tumor cells within a microenvironment through glycan-binding receptors–lectins (5). Glycans are oligosaccharide structures presented on protein and lipids. Endogenous lectins expressed on immune cells and other cells in the stroma, facilitate cell-interactions, -adhesions, thereby contributing to homeostasis. During malignancy, glycans on tumor cells are involved in invasiveness, metastasis, and immune suppression (6–8).

Several families of cell adhesion molecules including cadherins, integrins, junctional-adhesion molecules, and selectins are altered during tumorigenesis. This mini review addresses the role of cell adhesion and glycan-mediated interactions during metastasis and tumor-induced immune suppression in the context of altered glycosylation as a ubiquitous characteristic of cancer progression with a focus on integrins, selectins, and siglecs.

## Selectins Contribute to Cancer Progression

Selectins are vascular cell adhesion receptors present on leukocytes, endothelial cells, and platelets that bind to glycans. The physiological function of selectins is to facilitate the initial tethering of leukocytes at inflammatory sites or secondary lymphoid organs or hemostasis (9, 10). There is accumulating evidence for the involvement of selectins in pathophysiological processes, including cancer metastasis (11, 12). There are three members of the selectin family: P-selectin expressed on activated platelets and endothelial cells, L-selectin present on leukocytes, and E-selectin expressed on activated endothelial cells (10). Upon activation, P-selectin is rapidly presented on the surface of activated endothelial cells or platelets through exocytosis of storage granules. E-selectin is present only on activated endothelial cells and its expression is regulated on a transcription level. L-selectin is constitutively expressed on most subsets of leukocytes.

Selectins are C-type lectins that bind to properly modified glycan ligands, carrying terminal sLe^x^ or sLe^a^ structures. Selectin binding to glycans usually requires a protein scaffold that presents selectin ligands in clusters (13). The best characterized selectin ligand is P-selectin glycoprotein ligand 1 (PSGL-1) (9, 10). All three selectins bind to PSGL-1 that is mostly expressed in leukocytes. In addition, selectins binds to these glycan moieties carried on several cell surface proteins, such as CD44, E-selectin ligand-1, CD43, CD34, or addressins with a variable specificity (14).

There is compelling experimental and clinical evidence for the enhanced expression of sLe^x^ and sLe^a^ to correlate with poor prognosis due to enhanced metastasis in tumors of gastric, pancreatic, colon, prostate, renal, lung, and melanoma cancers (4, 15). Enhanced expression of selectin ligands is linked to increased activities of glycosyltransferases, responsible for the terminal synthesis, sialyltransferases, and fucosyltransferases. Major carriers of selectin ligands are mucins that are heavily O-glycosylated (16). MUC1, MUC2, MUC4, and MUC16 are mucins associated with cancer progression, whereas MUC16 is also used for cancer diagnostics. However, the spectrum of selectin ligands on tumor cells is rather broad, encompassing glycolipids, proteins, and glycosaminoglycans (4).

**Figure 1:**
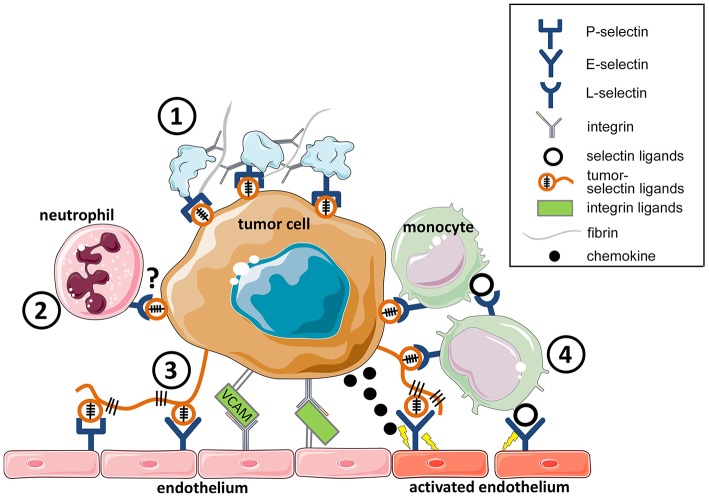
Cell adhesion facilitates tumor cell survival in the circulation and tumor cell extravasation. Tumor cells in the circulation interact through selectins and integrins with blood constituents (platelets, leukocytes, and endothelial cells). **(1)** platelet-tumor cell aggregate formation is mediated by both P-selectin and integrins through fibrin and fibrinogen. **(2)** The survival of circulating tumor cells is further enhanced by aggregation with neutrophils that promote tumor cell proliferation (17). Whether L-selectin or integrins mediate these interactions remains to be determined. **(3)** Tumor cell interaction with the endothelium, leading to adherence, is mediated by P-and E-selectins, and tumor cell firm adhesion is facilitated by integrins, and their interaction, for example with VCAM-1 on tumor cells. **(4)** Tumor cell-endothelial interaction directly or facilitated by monocytes, contribute to the initiation of tumor cell extravasation. This process is dependent both on E-selectin and integrin engagement.

During the hematogenous phase of metastasis, tumor cells carrying selectin ligands (Figure 1) enter the blood circulation and encounter selectins on platelets, leukocytes, and on the endothelium (14). Tumor cells in the circulation are often associated with platelets that protects them from the immune system (18), and enables tumor cell seeding in distant organs (19). The absence of P-selectin abrogates platelet-tumor cell aggregation and consequently attenuates metastasis (20). In addition to P-selectin, platelets also express CD40 on their surfaces, which upon binding to the CD40 ligand accelerate endothelial inflammation and atherosclerosis (21). Platelet-leukocyte aggregate formation resulted in a release of IL-1β by leukocytes. CD40 deficiency in the blood compartment attenuated experimental lung metastasis (22), indicating a potential involvement of platelet CD40 in cancer progression. Tumor cell arrest in the vasculature induces local activation of the endothelium and results in expression of E-selectin and chemokines (12, 23). Chemokine-driven recruitment of inflammatory monocytes to metastasizing cells was shown to promote metastasis in different cancers [reviewed in (24)]. E-selectin has been shown to promote tumor cell adhesion and thereby metastatic dissemination (14, 25, 26). Enhanced expression of E-selectin ligands on human breast cancer cells, such as CD44, promotes homing to the microvascular endothelium and metastasis (27).

Selectins not only mediate tumor cell adhesion but actively contribute to the formation of a metastasis niche (28, 29). Effective tumor cell extravasation in the lungs requires engagement of E-selectin on activated endothelial cells, which is essential for the loosening of endothelial VE-cadherin junctions (28). E-selectin promotes the recruitment of inflammatory monocytes, Ly6C^hi^ cells that facilitate transendothelial migration of tumor cells (Figure 1). The opening of endothelial junctions was shown to be dependent on the Src kinase pathway that induces E-selectin expression (30). Recently, E-selectin in the bone marrow vascular niche was shown to promote metastasis by inducing mesenchymal-epithelial transition of breast cancer cells through the activation of Wnt signaling (29). Tumor cell expression of fucosyltransferase-7, required for E-selectin ligand formation, is essential for the formation of bone metastasis.

L-selectin-mediated recruitment of leukocytes promotes both tumor cell extravasation and the formation of a metastatic niche (28, 31, 32). Tumor-induced endothelial activation is associated with selectin ligand accumulation required for the L-selectin dependent recruitment of myeloid cells (31), which were later identified to be inflammatory monocyte Ly6C^hi^ cells (33, 34). Interestingly, the presence of selectin ligands on leukocytes is also required for their effective recruitment to the metastatic sites (34). L-selectin facilitates the recruitment of T-cells to the lymph nodes. An engagement of a T cell receptor leads to the shedding of L-selectin from the cell surface of T cells (35, 36). Notably, cytotoxic/memory T cells, with L-selectin expression, better controlled tumor growth (37, 38). Sustained L-selectin expression on NK cells was shown to control tumor progression (39). Recently, the role of L-selectin on T cells was investigated in a transgenic mouse model that expressed non-cleavable L-selectin (40). T cells with constitutive expression of L-selectin suppressed lung metastasis. However, how L-selectin on T and NK cells contributes to the immune suppressive activity during metastasis remains to be defined.

## Siglec-Mediated Immune Suppression in Cancer

The cell surface glycans of mammalian cells commonly terminate with sialic acid (41). These sialylated structures, also called sialoglycan can engage various endogenous receptors including sialic acid-binding immunoglobulin-like lectins (Siglecs) (7, 42–44). Siglecs are mostly inhibitory receptors with an extracellular part that contains an N-terminal carbohydrate recognition domain (CRD) and a variable number of C2 domains (44). The intracellular part of inhibitory Siglecs contains ITIM or ITIM-like structures mediating immune inhibition (7, 42–44). Activating Siglecs have a positively charged amino acid in the transmembrane domain that mediates interaction with DAP12 upon Siglec engagement (7, 42–44). In humans, 14 different, functionally active Siglecs were identified. The conserved Siglecs Siglec-1 (sialoadhesin), CD22 (Siglec-2), Siglec-4 (MAG), and Siglec-15 have orthologs across different mammalian species (45, 46). CD33-related Siglecs, however, have undergone rapid evolutionary adaptation (45, 46). This subfamily includes CD33 (Siglec-3), Siglec-5, Siglec-6, Siglec-7, Siglec-8, Siglec-9, Siglec-10, and Siglec-11 (45, 46). In mice, no direct orthologs of human CD33-related Siglecs can be found, but functional paralogues with similar expression patterns can be defined (47).

Siglec receptors are predominantly expressed on immune cells (7, 42–44). Inhibitory Siglec receptors can modulate immune cell activation by recruitment of SHP1 and SHP2 phosphatase upon binding to sialoglycans (7, 42–44). Binding-specificity varies between different Siglecs. While Siglec-9 has quite a broad binding spectrum (47, 48), Siglec-8 binds a rather restricted set of sialoglycans which also contain sulfate groups (49, 50). The binding spectrum and the expression patterns determine the function of Siglecs.

Recent evidence has shown that tumor cells can also engage the sialoglycan-Siglec axis to evade immune control (7, 47, 51–56). In many cancer types, the glycocalyx and also the tumor microenvironment are characterized by an enhanced presence of sialoglycans due to changes in sialic acid-modifying enzymes including sialic acid synthesis genes, transporters, sialyltransferases, and sialidases (57–59). Moreover, enzymes such as O-acetylases can directly modify the sialic acid residues (57–59). This upregulation of sialoglycans in some cancers is termed hypersialylation, which is quite heterogenous between different cancer types but also within a specific cancer type. In lung cancer, we have observed considerable heterogeneity of sialoglycan ligands for Siglec-7 and Siglec-9 (51). Similar observations were made in melanoma samples (52). How sialylation differs within a single cancer patient and how hypersialylation evolves during different treatments and during cancer progression over time remains to be determined.

**Figure 2:**
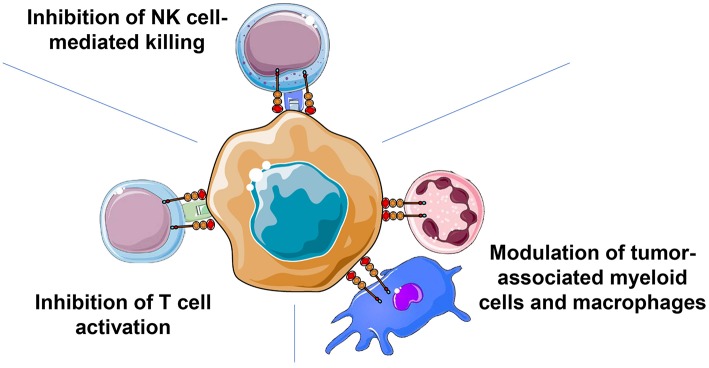
The sialoglycan-Siglec glyco-immune checkpoint involves cells of the innate and the adaptive immune response. Cancer-associated sialoglycans on the surface of tumor cells but also within the tumor microenvironment can mediate immune evasion by engaging Siglec receptors on cells of the innate (NK cells, myeloid cells, and macrophages) and the adaptive (T cells) immune system. Inhibitory Siglec receptors, for example Siglec-9, can inhibit T cell activation by modulating signaling of the T cell receptor. Similarly, NK cell activation and tumor cell killing can be reduced by inhibitory Siglecs such as Siglec-7 and Siglec-9. Interactions of cancer-associated sialoglycans can also regulate myeloid cells and tumor-associated macrophages by influencing the polarization of TAMs and potentially influencing macrophage-mediated phagocytosis via inhibitory Siglec receptors.

The increased density of sialoglycans can lead to engagement of inhibitory Siglec receptors on immune cells and modulate the immune response to cancer (Figure 2). Both innate and adaptive immune cells can be regulated by the sialoglycan-Siglec checkpoint. Human NK cells express inhibitory Siglec-7 and some subpopulations of NK cells also express Siglec-9 (54, 55). Engagement of Siglec-7 and/or Siglec-9 can inhibit NK cell-mediated tumor cell killing *in vitro* (54, 55). The introduction of a synthetic sialoglycan polymer into the glycocalyx of target cells led to a significant decrease in the NK cell-mediated killing of cells lacking MHC I expression and a reduced antibody-dependent cellular cytotoxicity (54). Antibodies blocking Siglec-7 or Siglec-9 resulted in increased tumor cell killing (55). In addition, sialic acid-dependent NK cell inhibition was also observed in a humanized mouse model (55). Macrophage polarization is also influenced by a sialoglycan-Siglec pathway (47, 56). Alternative M2 polarized macrophages produce cytokines suppressing anti-cancer immunity, secrete pro-angiogenic factors, enhance tumor cell invasion, and thereby promote cancer progression (60, 61). Binding of sialylated, cancer-associated MUC1 to Siglec-9 led to a polarization to M2 macrophages *in vitro* (56). However, studies in Siglec-E deficient mice showed a propensity of Siglec-E deficient macrophages to polarize to M2 macrophages (47). Macrophages express various Siglecs including Siglec-3, Siglec-5/-14, Siglec-7, Siglec-9, and Siglec-10 with some overlapping binding spectra (7, 42–44). The exact function of sialoglycan-Siglec interactions on the influence of pro- and anti-tumorigenic effects of tumor-associated macrophages certainly require further studies. For example, Siglec receptors could also act as potential “don't eat me” signals that inhibit macrophage-mediated phagocytosis (62). Conserved Siglec-15 was identified in a screening of surface markers on antigen-presenting cells that could inhibit T cell activation (63). Antibodies against Siglec-15 tested in a murine tumor model led to enhanced anti-cancer immunity (63). Antibodies were humanized and early clinical trials are being planned.

Recent work provided evidence that Siglec receptors are expressed on platelets in both humans and mice (64, 65). Engagement of Siglec-9 or Siglec-E on platelets increased the infectivity of group B streptococci by modulation of platelet activation (64). One could hypothesize that interactions of tumor cell-sialoglycans could also modulate platelet activation and influence metastatic progression.

Two recent studies have found that the sialoglycan-Siglec glyco-immune checkpoint influences activation of tumor-infiltrating lymphocytes (TILs), particularly cytotoxic CD8+ T cells (51, 52). We have found that TILs upregulate different inhibitory CD33-related Siglecs, predominantly Siglec-9 in patients with non-small cell lung cancer, colorectal cancer, epithelial ovarian cancer and melanoma (51, 52). Healthy peripheral blood T cells, however, were not expressing these inhibitory receptors, as described earlier (51, 52). Siglec-E was upregulated on tumor-infiltrating T cells in murine tumor models (51). Inhibition of the sialoglycan-Siglec axis with blocking antibodies or genetic models enhances T cell-mediated anti-cancer immunity *in vitro* and *in vivo* (51, 66, 67). These results directly implicate that Siglec-9 is a new target that can improve anti-tumoral T cell activation.

Targeting the sialoglycan-Siglec glyco-immune checkpoint can be achieved by using Siglec-blocking antibodies. Another approach is the reduction of the ligand-density by targeting sialoglycans. Using a sialic acid mimetic that inhibits intratumoral sialoglycan production led to enhanced T cell-mediated anti-tumor immunity (68). Similar findings were observed with tumor cell lines with defects in sialic acid biosynthesis (51, 69). An elegant therapeutic approach is the use of sialidases fused to tumor-targeting antibodies that, upon systemic application, mediate hyposialylation of the tumor microenvironment. Xiao et al. have used the anti-HER2 antibody trastuzumab fused with a bacterial sialidase which was shown to increase tumor cell killing *in vitro* (70) and is currently being tested in pre-clinical mouse models.

## Integrins During Tumor Cell Dissemination and Metastatic Colonization

Integrin binding to the components of extracellular matrix (ECM) enables the cell “to sense” the environment and to activate intracellular signaling, which modulates cellular behaviors including survival, proliferation, and migration; thereby sustaining homeostasis. During malignancy, altered expression of integrins together with the loss of cell polarity profoundly changes the cell signaling, which alters oncogenic activity, cell stemness, epithelial plasticity, and angiogenesis [reviewed elsewhere (2, 71, 72)].

The integrins comprise a family of heterodimeric α/β integrin receptors, which facilitates contacts with components of the extracellular matrix (ECM) and in some cases with adhesion receptors on other cells. A particular integrin receptor with a preference for a specific ligand defines a cell based on the recognition of ECM (e.g., fibronectin, laminin, or collagen). Two receptors, vascular cell adhesion molecule 1 (VCAM1), and intracellular cell adhesion molecules (ICAMs) serve as cell surface receptors for integrins. Integrin-based adhesion of a cell facilitates intracellular adaptor proteins that recruit kinases, for example focal adhesion kinase (FAK) or Src family kinases; and induces signal transduction (2, 72, 73). Conversely, external signals (growth factors or cytokines) may change the intracellular recruitment of integrins resulting in the modulation of integrin affinity. Integrins can initiate pro-survival but also pro-apoptotic signals (74–76).

A variety of integrins expressed on tumors that originate from epithelial cells, typically facilitate cell adhesion to the basement membrane. Tumor cell surface expression of integrins can vary widely, but is generally associated with the enhanced presence of αvβ3, αvβ6, α5β1, α6β4 which correlates with the metastatic progression in melanoma, prostate, pancreatic, colon, lung, and breast cancers [reviewed in (73)]. Importantly, integrins within the tumor microenvironment present on endothelial cells, leukocytes, platelets, and other cells of the stroma significantly modulate tumor progression and particularly metastasis.

During the hematogenous phase of metastasis tumor induced platelet activation and the formation of platelet-and fibrin-rich tumor cell thrombi (Figure 1), are mediated both by integrins and selectins (18, 77–80). In particular, susceptibility to metastasis is associated with tumor cell-derived deposition of certain ECM proteins, such as tenascin C (TNC) in the metastatic niche. TNC is a ligand for β1- and β3-integrin and its accumulation in the lungs promotes tumor cell outgrowth and metastasis (81). Osteosarcoma metastasis to the lungs is dependent on TNC expression and the respective expression a receptor on tumor cell α9β1 integrin (82). The trabecular bone rich in TNC was shown to promote prostate homing of cancer metastasis through α9β1 integrin (83). Metastatic lung colonization was associated with an induction of stromal periostin expression that is recognized by β1 and β5 integrins on tumor cells (84).

Integrins also contribute to the formation of a tumor microenvironment. The systemic absence of β4 integrin resulted in the attenuation of tumor growth due to impaired angiogenesis (85). Tumor-induced lymphangiogenesis promotes metastasis to the lymph node through the activation of α4β1 integrin on lymphatic endothelium, which binds to VCAM1-positive tumor cells (86). In addition, myeloid cells expressing α4β1 integrins accumulate on the tumor-activated endothelium and promote metastasis (87). Antagonist of α4β1 integrin blocked the recruitment of myeloid cells and thereby angiogenesis and tumor growth. The aberrant expression of VCAM-1 on dormant tumor cells in bone marrow was shown to recruit α4β1-expressing osteoclast progenitors during bone metastasis (88). It has recently been demonstrated that chemoresistant disseminated tumors occupying the perivascular niche, interacts through β1-integrin with VCAM-1 on endothelial cells (89). The inhibition of β1-integrin or VCAM-1 sensitizes tumor cells to chemotherapy, making integrin inhibition a viable therapeutic approach to prevent metastasis.

Tumor cell expression of αvβ3 of α4β1 integrins is linked to bone metastasis, where they support tumor cell adhesion to ECM proteins such as osteopontin or type I collagen (90). Melanoma cells expressing α4β1 integrins metastasized to lymph nodes (Figure 1) by binding to VCAM-1 on lymphatic endothelial cells (91). Tumor cell α_3_β_1_ integrin facilitates metastasis by binding to the exposed basement membrane protein laminin-5 in the lungs, thereby promoting tumor cell arrest and the onset of outgrowth (92, 93). Recently, integrins on tumor-derived exosomes were shown to drive the organotropism of tumor metastasis (94). Tumor-derived exosomes carrying α6β4 integrin, target a laminin-rich lung microenvironment, where they induce the accumulation of pro-inflammatory factors required for the promotion of tumor cell seeding and metastasis. While tumor cells do not express leukocyte-specific β2 integrins, for example LFA-1, tumor cells that express ICAM-1 facilitate their adherence to leukocytes, particularly neutrophils, through β2 integrins, which in turn bind to the endothelium, thereby promoting metastasis (95, 96). The role of integrins during cancer is dependent on cues in a tissue context-dependent manner.

Altered glycosylation of integrins on tumor cells modulate the intracellular signaling and cell adhesion (97, 98). An overexpression of branched β1,6-N-acetylglucosamine (GlcNAc) on N-glycans, which is catalyzed by a GnT-V enzyme, is associated with poor prognosis (99), while a knock-down of GnT-V in breast carcinoma cells result in reduced invasiveness (100). Increased branched N-glycans on α3β1 integrins in B16 mouse melanoma correlated with lung metastasis (101). Galectins are a family of β-galactoside-binding soluble lectins expressed by tumor cells. The N-glycan on integrin induces the complex formation of α6β4 integrin/EGFR/ galectin-3 which promotes integrin clustering and cell migration and proliferation (102, 103). Galectin-3 induced αvβ3 integrin-mediated clustering was shown to cause tumor growth and drug resistance (104). Mechanistically, unbound αvβ3 integrin on tumor cells recruits KRAS to the cell membrane and activates downstream signaling through the NF-κB pathway and promotes pancreatic carcinoma. In hepatocellular carcinoma metastasis, O-glycosylation of β1 integrin influences tumor migration (105). Terminal sialylation on N-glycans and O-glycans of glycoproteins is frequently observed in cancer (44, 59, 106). Hypersialylation detected in colon, stomach, and ovarian cancers has been linked to an enhanced expression of α2,6-sialyltransferase (ST6Gal-I) and is identified as a marker of poor prognosis (106). In colon carcinoma, enhanced sialylation of β1 integrin facilitates adhesion to collagen I and the migration of tumor cells (107). Accordingly, inhibition of ST6Gal-I expression blocks collagen binding and tumor cell migration. Desialylation of O-glycans by sialidase NEU1 suppresses colon-tumor cell adhesion to laminin, tyrosine phosphorylation of integrin β4 and metastasis (108). However, in breast cancer cells the α2,6 hypersialylation of β1 integrin decreased the adhesion but did not affect invasiveness of these cells (109). These data indicate that glycosylation of integrins modulates adhesion, migration, and signaling of metastatic cells.

## Conclusions and Future Directions

Alterations in cell adhesion and cell-cell interactions of tumor cells are inherently linked to many processes associated with immune evasion and metastasis that go beyond the scope of this review. The very nature of adhesion receptors on cells to interrogate signals from outside makes the tumor microenvironment a crucial factor during immune evasion and metastasis. The biology of cell adhesion during cancer progression remains complex mainly due to: (a) several receptors likely act in parallel during any metastasis; (b) cell surface changes are linked to tumor heterogeneity; (c) diverse tumor glycosylation affect both receptors and ligands. Nevertheless, the potential to target cell adhesion mechanisms for tumor therapies is continuously being explored. For instance, fusion of a IL-2 cytokine with an Fc part of an antibody targeting RGD sequence of an integrin demonstrated promising results in mouse models when applied in combination with a PD-1 checkpoint blockade inhibitor (110). Another study has demonstrated that fucosylated nanoparticles can target irradiation-activated vasculature of the tumor, associated with enhanced P-selectin expression (111). Altered tumor glycosylation is a common culprit that contributes to tumor cell dissemination and immune suppression. Thus, further efforts to “edit” the tumor glycosylation landscape using sialidase and thereby changing the Siglec immune responsiveness or metastasis holds great potential in clinical applications (70). Nevertheless, further understanding of the tumor microenvironment is a prerequisite for designing an intervention on cell adhesion mechanisms that will be likely used in combination either with standard- or immune-therapy.

## Author Contributions

All authors listed have made a substantial, direct and intellectual contribution to the work, and approved it for publication.

### Conflict of Interest Statement

HL received travel grants and consultant fees from Bristol-Myers Squibb (BMS) and Merck, Sharp, and Dohme (MSD), and Roche. HL received research support from BMS and Palleon Pharmaceuticals. The remaining author declares that the research was conducted in the absence of any commercial or financial relationships that could be construed as a potential conflict of interest.
